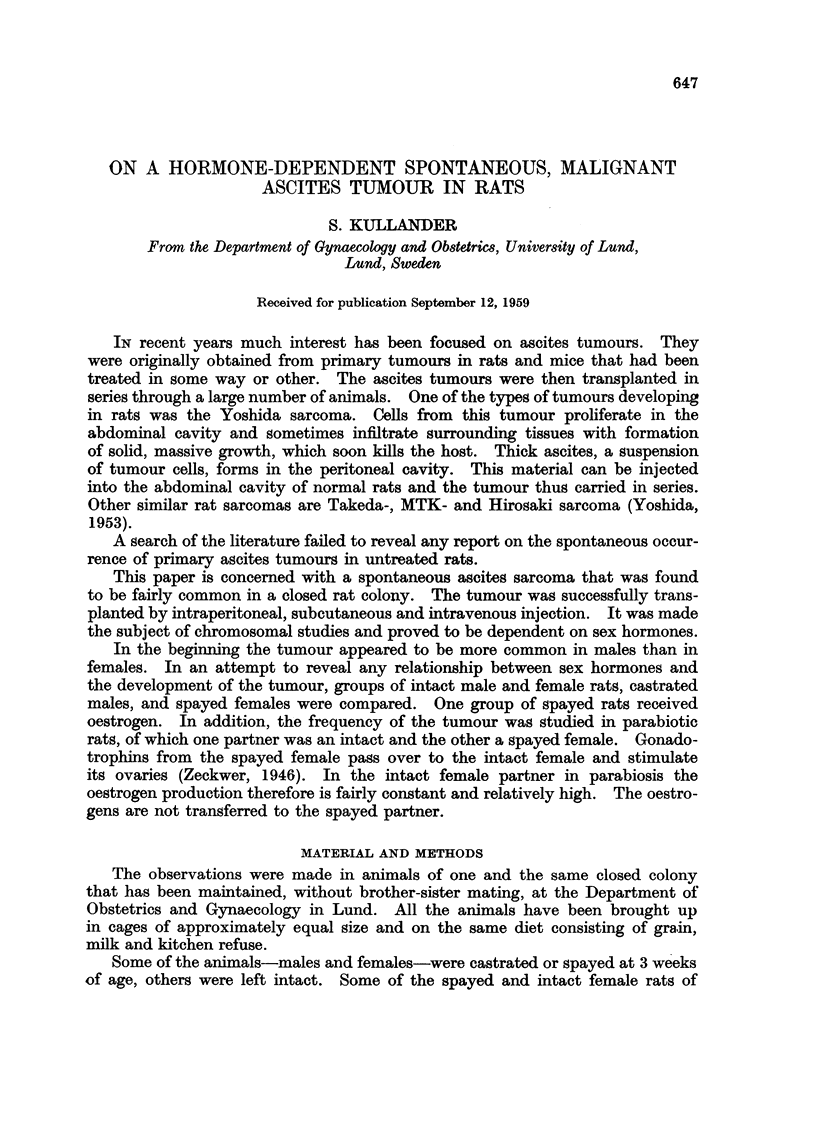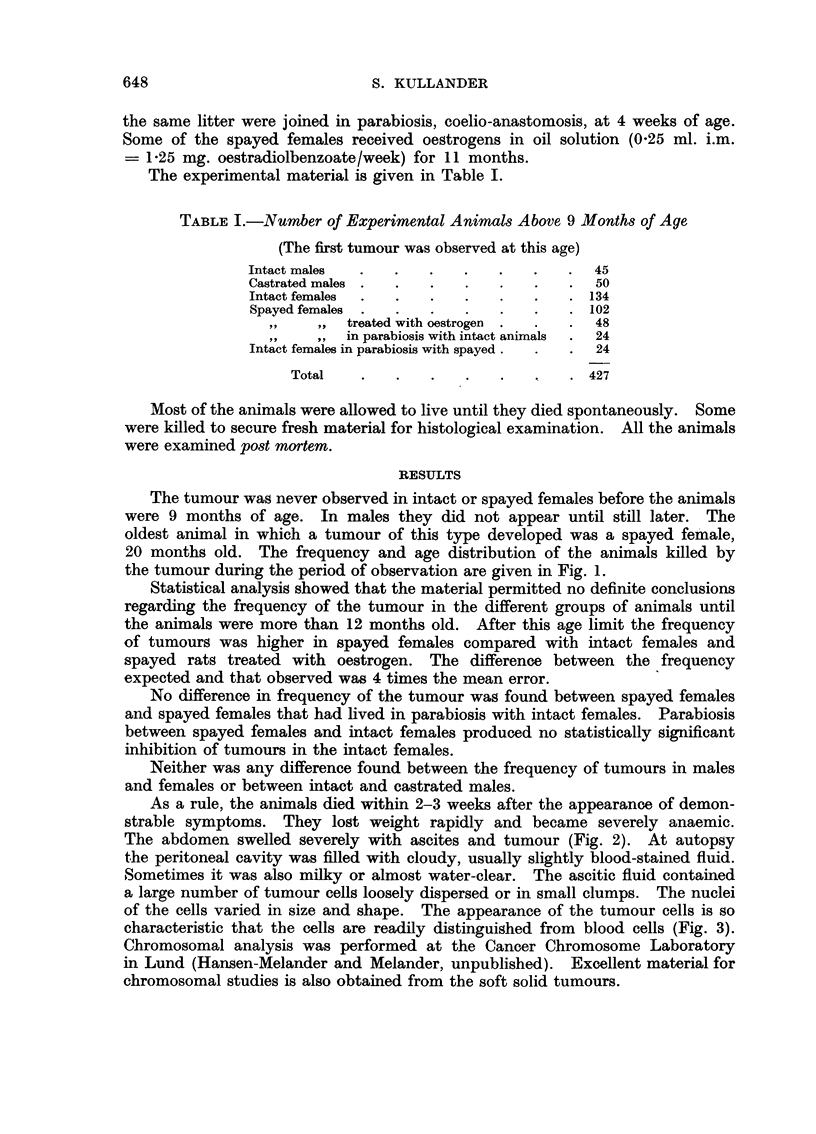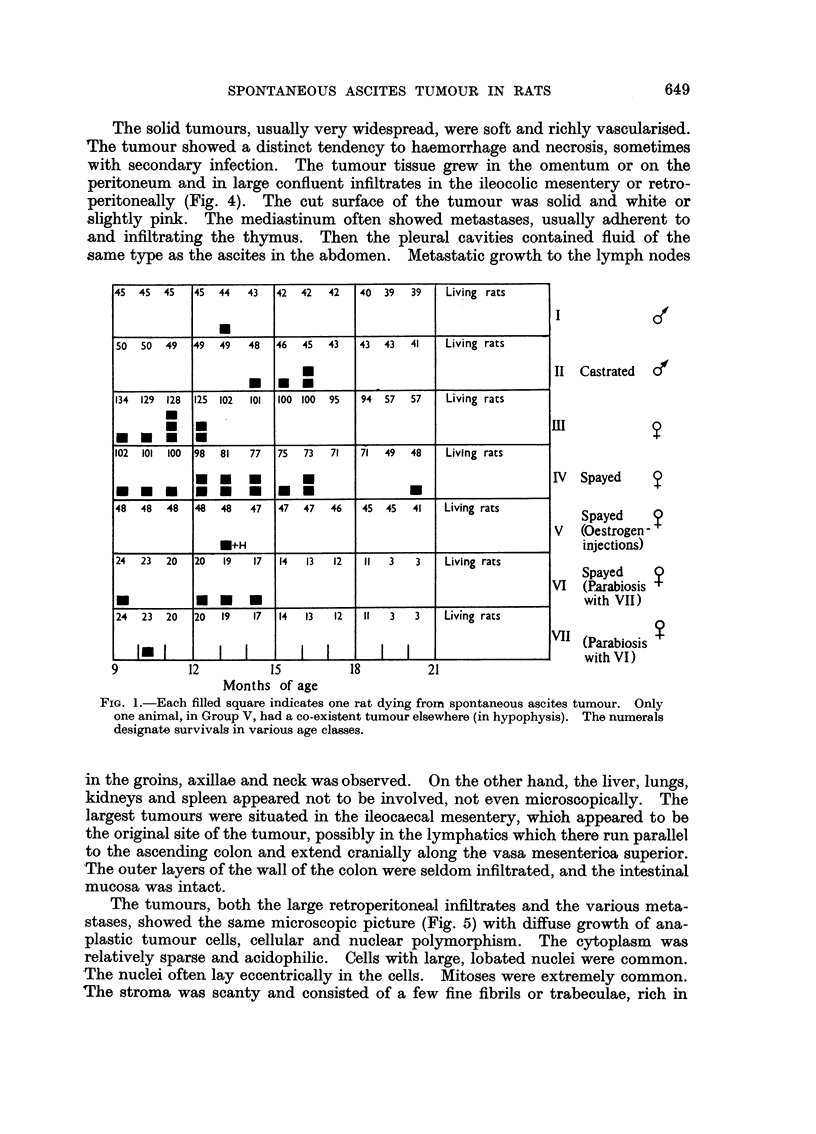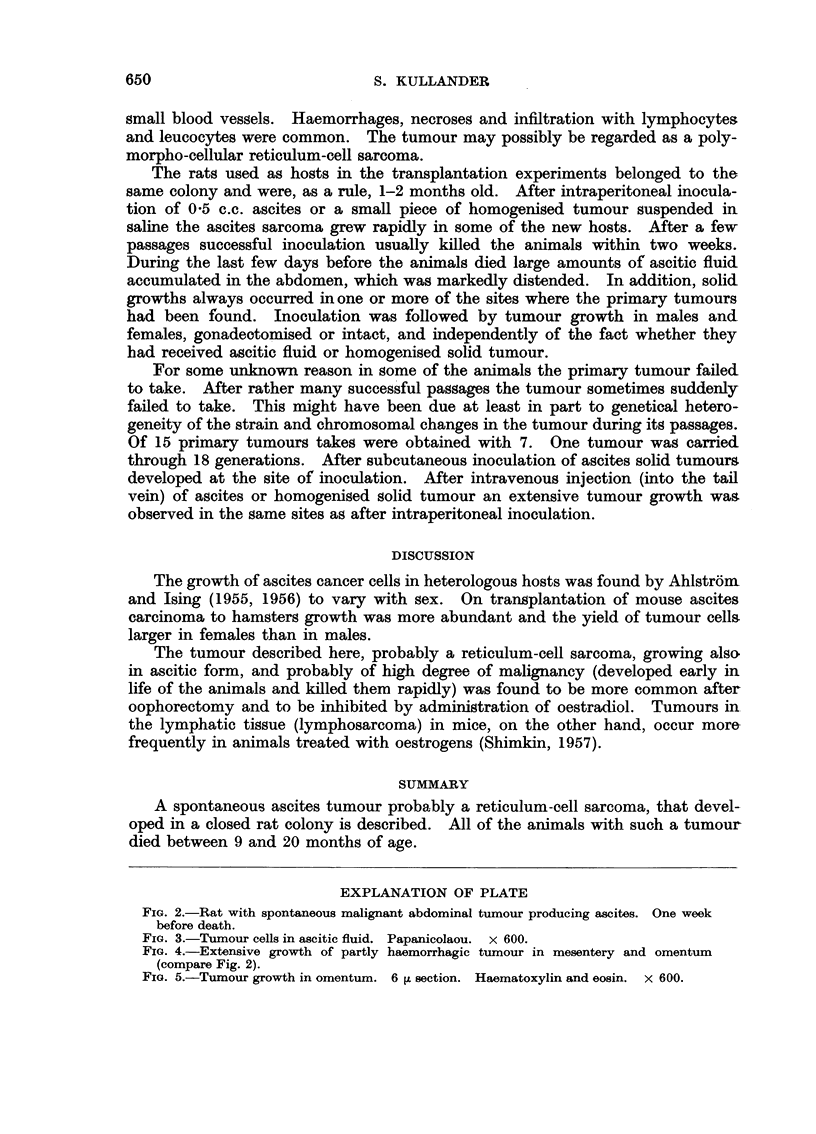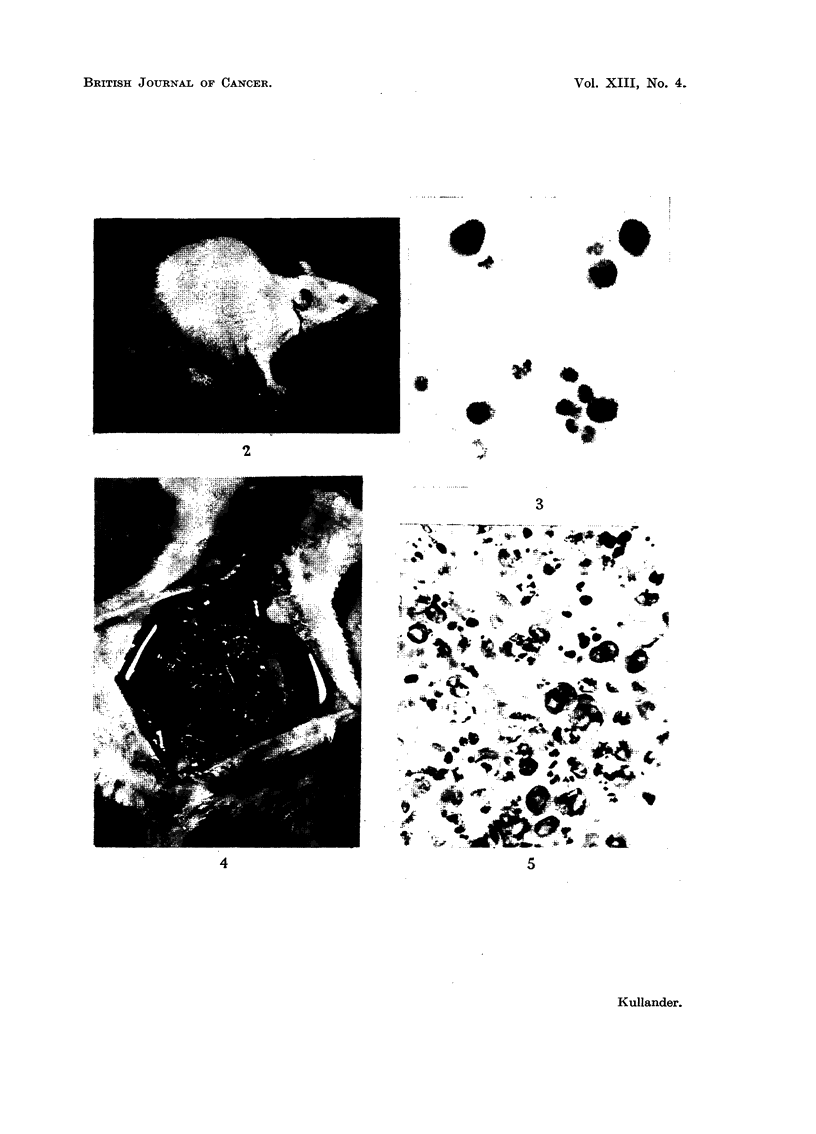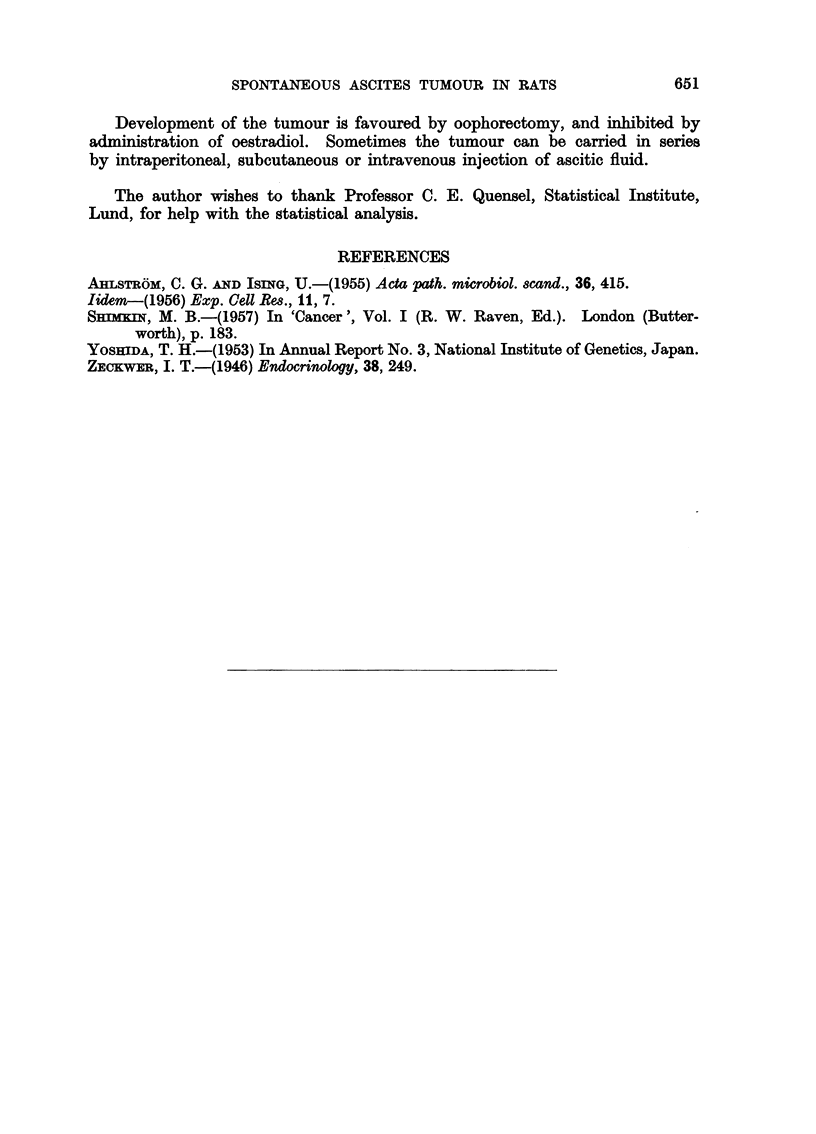# On a Hormone-Dependent Spontaneous, Malignant Ascites Tumour in Rats

**DOI:** 10.1038/bjc.1959.71

**Published:** 1959-12

**Authors:** S. Kullander

## Abstract

**Images:**


### ON A HORMONE-DEPENDENT SPONTANEOUS, MALIGNANT ASCITES TUMOUR IN RATS

S. KULLANDER

*From the Department of Gynaecology and Obstetrics, University of Lund, Lund, Sweden*

Received for publication September 12, 1959

IN recent years much interest has been focused on ascites tumours. They were originally obtained from primary tumours in rats and mice that had been treated in some way or other. The ascites tumours were then transplanted in series through a large number of animals. One of the types of tumours developing in rats was the Yoshida sarcoma. Cells from this tumour proliferate in the abdominal cavity and sometimes infiltrate surrounding tissues with formation of solid, massive growth, which soon kills the host. Thick ascites, a suspension of tumour cells, forms in the peritoneal cavity. This material can be injected into the abdominal cavity of normal rats and the tumour thus carried in series. Other similar rat sarcomas are Takeda-, MTK- and Hirosaki sarcoma (Yoshida, 1953).

A search of the literature failed to reveal any report on the spontaneous occurrence of primary ascites tumours in untreated rats.

This paper is concerned with a spontaneous ascites sarcoma that was found to be fairly common in a closed rat colony. The tumour was successfully transplanted by intraperitoneal, subcutaneous and intravenous injection. It was made the subject of chromosomal studies and proved to be dependent on sex hormones.

In the beginning the tumour appeared to be more common in males than in females. In an attempt to reveal any relationship between sex hormones and the development of the tumour, groups of intact male and female rats, castrated males, and spayed females were compared. One group of spayed rats received oestrogen. In addition, the frequency of the tumour was studied in parabiotic rats, of which one partner was an intact and the other a spayed female. Gonadotrophins from the spayed female pass over to the intact female and stimulate its ovaries (Zeckwer, 1946). In the intact female partner in parabiosis the oestrogen production therefore is fairly constant and relatively high. The oestrogens are not transferred to the spayed partner.

### MATERIAL AND METHODS

The observations were made in animals of one and the same closed colony that has been maintained, without brother-sister mating, at the Department of Obstetrics and Gynaecology in Lund. All the animals have been brought up in cages of approximately equal size and on the same diet consisting of grain, milk and kitchen refuse.

Some of the animals—males and females—were castrated or spayed at 3 weeks of age, others were left intact. Some of the spayed and intact female rats of the same litter were joined in parabiosis, coelio-anastomosis, at 4 weeks of age. Some of the spayed females received oestrogens in oil solution (0·25 ml. i.m. = 1·25 mg. oestradiolbenzoate/week) for 11 months.

The experimental material is given in Table I.

TABLE I.—*Number of Experimental Animals Above* 9 *Months of Age*

(The first tumour was observed at this age)

| | |
|---|---|
| Intact males | 45 |
| Castrated males | 50 |
| Intact females | 134 |
| Spayed females | 102 |
| ,, ,, treated with oestrogen | 48 |
| ,, ,, in parabiosis with intact animals | 24 |
| Intact females in parabiosis with spayed | 24 |
| Total | 427 |

Most of the animals were allowed to live until they died spontaneously. Some were killed to secure fresh material for histological examination. All the animals were examined *post mortem.*

### RESULTS

The tumour was never observed in intact or spayed females before the animals were 9 months of age. In males they did not appear until still later. The oldest animal in which a tumour of this type developed was a spayed female, 20 months old. The frequency and age distribution of the animals killed by the tumour during the period of observation are given in Fig. 1.

Statistical analysis showed that the material permitted no definite conclusions regarding the frequency of the tumour in the different groups of animals until the animals were more than 12 months old. After this age limit the frequency of tumours was higher in spayed females compared with intact females and spayed rats treated with oestrogen. The difference between the frequency expected and that observed was 4 times the mean error.

No difference in frequency of the tumour was found between spayed females and spayed females that had lived in parabiosis with intact females. Parabiosis between spayed females and intact females produced no statistically significant inhibition of tumours in the intact females.

Neither was any difference found between the frequency of tumours in males and females or between intact and castrated males.

As a rule, the animals died within 2–3 weeks after the appearance of demonstrable symptoms. They lost weight rapidly and became severely anaemic. The abdomen swelled severely with ascites and tumour (Fig. 2). At autopsy the peritoneal cavity was filled with cloudy, usually slightly blood-stained fluid. Sometimes it was also milky or almost water-clear. The ascitic fluid contained a large number of tumour cells loosely dispersed or in small clumps. The nuclei of the cells varied in size and shape. The appearance of the tumour cells is so characteristic that the cells are readily distinguished from blood cells (Fig. 3). Chromosomal analysis was performed at the Cancer Chromosome Laboratory in Lund (Hansen-Melander and Melander, unpublished). Excellent material for chromosomal studies is also obtained from the soft solid tumours.

The solid tumours, usually very widespread, were soft and richly vascularised. The tumour showed a distinct tendency to haemorrhage and necrosis, sometimes with secondary infection. The tumour tissue grew in the omentum or on the peritoneum and in large confluent infiltrates in the ileocolic mesentery or retroperitoneally (Fig. 4). The cut surface of the tumour was solid and white or slightly pink. The mediastinum often showed metastases, usually adherent to and infiltrating the thymus. Then the pleural cavities contained fluid of the same type as the ascites in the abdomen. Metastatic growth to the lymph nodes

FIG. 1.—Each filled square indicates one rat dying from spontaneous ascites tumour. Only one animal, in Group V, had a co-existent tumour elsewhere (in hypophysis). The numerals designate survivals in various age classes.

in the groins, axillae and neck was observed. On the other hand, the liver, lungs, kidneys and spleen appeared not to be involved, not even microscopically. The largest tumours were situated in the ileocaecal mesentery, which appeared to be the original site of the tumour, possibly in the lymphatics which there run parallel to the ascending colon and extend cranially along the vasa mesenterica superior. The outer layers of the wall of the colon were seldom infiltrated, and the intestinal mucosa was intact.

The tumours, both the large retroperitoneal infiltrates and the various metastases, showed the same microscopic picture (Fig. 5) with diffuse growth of anaplastic tumour cells, cellular and nuclear polymorphism. The cytoplasm was relatively sparse and acidophilic. Cells with large, lobated nuclei were common. The nuclei often lay eccentrically in the cells. Mitoses were extremely common. The stroma was scanty and consisted of a few fine fibrils or trabeculae, rich in small blood vessels. Haemorrhages, necroses and infiltration with lymphocytes and leucocytes were common. The tumour may possibly be regarded as a polymorpho-cellular reticulum-cell sarcoma.

The rats used as hosts in the transplantation experiments belonged to the same colony and were, as a rule, 1–2 months old. After intraperitoneal inoculation of 0·5 c.c. ascites or a small piece of homogenised tumour suspended in saline the ascites sarcoma grew rapidly in some of the new hosts. After a few passages successful inoculation usually killed the animals within two weeks. During the last few days before the animals died large amounts of ascitic fluid accumulated in the abdomen, which was markedly distended. In addition, solid growths always occurred in one or more of the sites where the primary tumours had been found. Inoculation was followed by tumour growth in males and females, gonadectomised or intact, and independently of the fact whether they had received ascitic fluid or homogenised solid tumour.

For some unknown reason in some of the animals the primary tumour failed to take. After rather many successful passages the tumour sometimes suddenly failed to take. This might have been due at least in part to genetical heterogeneity of the strain and chromosomal changes in the tumour during its passages. Of 15 primary tumours takes were obtained with 7. One tumour was carried through 18 generations. After subcutaneous inoculation of ascites solid tumours developed at the site of inoculation. After intravenous injection (into the tail vein) of ascites or homogenised solid tumour an extensive tumour growth was observed in the same sites as after intraperitoneal inoculation.

### DISCUSSION

The growth of ascites cancer cells in heterologous hosts was found by Ahlström and Ising (1955, 1956) to vary with sex. On transplantation of mouse ascites carcinoma to hamsters growth was more abundant and the yield of tumour cells larger in females than in males.

The tumour described here, probably a reticulum-cell sarcoma, growing also in ascitic form, and probably of high degree of malignancy (developed early in life of the animals and killed them rapidly) was found to be more common after oophorectomy and to be inhibited by administration of oestradiol. Tumours in the lymphatic tissue (lymphosarcoma) in mice, on the other hand, occur more frequently in animals treated with oestrogens (Shimkin, 1957).

### SUMMARY

A spontaneous ascites tumour probably a reticulum-cell sarcoma, that developed in a closed rat colony is described. All of the animals with such a tumour died between 9 and 20 months of age.

---

### EXPLANATION OF PLATE

FIG. 2.—Rat with spontaneous malignant abdominal tumour producing ascites. One week before death.

FIG. 3.—Tumour cells in ascitic fluid. Papanicolaou. × 600.

FIG. 4.—Extensive growth of partly haemorrhagic tumour in mesentery and omentum (compare Fig. 2).

FIG. 5.—Tumour growth in omentum. 6 μ section. Haematoxylin and eosin. × 600.

2

3

4

5

Kullander.

Development of the tumour is favoured by oophorectomy, and inhibited by administration of oestradiol. Sometimes the tumour can be carried in series by intraperitoneal, subcutaneous or intravenous injection of ascitic fluid.

The author wishes to thank Professor C. E. Quensel, Statistical Institute, Lund, for help with the statistical analysis.

### REFERENCES

AHLSTRÖM, C. G. AND ISING, U.—(1955) *Acta path. microbiol. scand.*, **36**, 415.
*Iidem*—(1956) *Exp. Cell Res.*, **11**, 7.
SHIMKIN, M. B.—(1957) In 'Cancer', Vol. I (R. W. Raven, Ed.). London (Butterworth), p. 183.
YOSHIDA, T. H.—(1953) In Annual Report No. 3, National Institute of Genetics, Japan.
ZECKWER, I. T.—(1946) *Endocrinology*, **38**, 249.